# Spreading Depolarization (SD): A Potential Underlying Mechanism in Functional Neurological Disorder (FND)

**DOI:** 10.1136/bcr-2025-266260

**Published:** 2025-07-31

**Authors:** Divine C. Nwafor, Cesarina Thohan, Chaitali Harge, C. William Shuttleworth, Andrew P. Carlson

**Affiliations:** 1 Department of Neurosurgery, University of Virginia, Charlottesville, VA, USA; 2 Department of Neurology, University of Virginia, Charlottesville, VA, USA; 3 Department of Neurosciences, University of New Mexico School of Medicine, Albuquerque, NM, USA

**Keywords:** FND, SD, Functional Neurological Disorder, Spreading Depolarization, Electroencephalography, Noninvasive Detection

## Abstract

Functional neurological disorder (FND) presents with neurological symptoms lacking a structural correlate and is thought to result from brain network dysfunction. Despite advances in understanding its pathophysiology, several knowledge gaps exist. Spreading depolarizations (SDs), waves of cortical electrical disturbance best known in migraine aura and implicated in conditions like stroke and traumatic brain injury, have not been investigated in the context of FND. We present a patient with recurrent episodes of limb weakness and speech disturbances initially diagnosed as FND. Years later, following a left Superficial temporal artery – middle cerebral artery bypass, she developed similar symptoms. Intraoperative electrocorticography revealed SDs coinciding with symptom onset, and treatment with SD inhibitors led to clinical improvement. While further studies are needed to establish causality, this case highlights SD’s as a plausible pathophysiological mechanism in a subset of FND patients and underscores the need for future research exploring their contribution to FND symptoms.

## 1. Background

Functional neurological disorder (FND) represents a complex condition characterized by neurological symptoms that are inconsistent with known structural brain lesions and cannot be explained by conventional neurological diseases. Historically referred to as conversion disorder, FND encompasses a wide spectrum of symptoms, including motor deficits (e.g., weakness, tremors), sensory disturbances (e.g., numbness, vision loss), cognitive impairments, and non-epileptic seizure-like episodes. Unlike traditional neurological disorders with clear structural correlates, FND is primarily diagnosed based on positive clinical signs and the exclusion of other causes.(1)

Emerging insights into FND pathophysiology highlight that it is not merely a psychological disorder but rather a multifactorial condition involving dysfunctions across brain networks responsible for sensory processing, motor control, emotion regulation, and cognitive functions. Neuroimaging studies have implicated altered connectivity between the prefrontal cortex, limbic system, and motor circuits, suggesting aberrant top-down modulation. Additionally, abnormalities in predictive coding—where the brain’s expectations disproportionately influence sensory perception—and impairments in the sense of agency have been proposed as plausible mechanisms.(1) This reflects a dynamic interplay between neural, psychological, and environmental factors, challenging the traditional mind-body dichotomy in FND.

The burden of FND on healthcare systems is substantial, with annual treatment costs exceeding $1 billion. Despite its prevalence, access to timely diagnosis and effective management remains limited due to stigma, misconceptions equating FND with malingering, and insufficient awareness among healthcare providers.(2, 3) While significant progress has been made in understanding the pathophysiology of FND, several mechanistic knowledge gaps remain.

In this report, we present the case of a woman in her late 60s whose recurrent neurological symptoms, initially attributed to FND, were later temporally correlated with spreading depolarizations (SDs). This case raises the possibility that SDs may serve as an underlying pathophysiological mechanism in a subset of patients diagnosed with FND, warranting further investigation.

## 2. Case Presentation

### I. History and Presentation

A woman in her late 60s with a history of coronary artery disease presented in 2022 with two hospitalizations for atypical neurological symptoms. The first episode occurred one week after a COVID-19 infection and urinary tract infection, with a second episode following one week later. CT and MRI of the brain showed no acute stroke, and a CT angiogram of the head and neck revealed moderate left cervical stenosis (<60%) and a distal occlusion of the left intracranial internal carotid artery (ICA) at the supraclinoid segment. Her symptoms were inconsistent, including right-sided weakness, trembling while ambulating, and complex speech disturbances such as stuttering, inability to use her phone, and transient mutism. Fluctuating facial droop was also reported over several days. Given the fluctuating nature of symptoms and absence of infarction, these episodes were deemed atypical for transient ischemic attack (TIA). She was initially started on dual antiplatelet therapy, which was later adjusted to direct oral anticoagulation (DOAC) plus aspirin after the second event. EEG testing was negative for epileptiform activity, and given the semiology of symptoms that included bilateral involvement and positive motor signs, she was referred to psychiatry for cognitive behavioral therapy (CBT) under the suspicion of functional neurological disorder (FND)/functional seizures.

For the next two years, she remained asymptomatic until she presented again with generalized weakness and right leg dyscoordination, occurring three days after a second COVID-19 infection. MRI at this time revealed bilateral deep punctate infarcts, more prominent in the left corona radiata. Diagnostic angiography confirmed left ICA occlusion and right ICA intracranial stenosis, measuring approximately 80%. She was discharged on medical management with aspirin and DOAC, having recovered to baseline, with plans for outpatient imaging follow-up.

Subsequent BOLD MRI with breath-holding challenge revealed severe steal from the left hemisphere and worsening deep white matter strokes in the corona radiata. Given the evidence of active ischemia, she was admitted for further management. Initial angioplasty of the right intracranial ICA resulted in minimal improvement, prompting a decision to proceed with a superficial temporal artery (STA) to middle cerebral artery (MCA) bypass. Informed consent was obtained for the placement of an electrocorticography (ECoG) recording electrode for short-term post-procedural monitoring. The procedure was completed successfully in a routine fashion (Figure 1A).

### II. Postoperative Course

Immediately post-procedure, the patient was neurologically intact. However, approximately 8 hours postoperatively, she developed new-onset speech disturbance, right lower face weakness, dysarthria, and generalized weakness. She was able to name some objects but not others. Emergent CTA ruled out acute stroke or bypass failure (Figure 1B). These symptoms fluctuated over the next several days, with the patient demonstrating limited vocalization but no true aphasia. She was able to communicate effectively through sign language. Given the atypical nature of her symptoms and reassuring imaging findings, concerns were raised about the possibility of recurrence of her prior functional neurological disorder.

Review of ECoG monitoring revealed spreading depolarizations (SDs) coinciding with symptom onset and recurring over several days (Figure 1A). To mitigate SDs, mean arterial pressures (MAPs) were optimized (>65 mmHg), and she was started on memantine (30 mg BID) and low-dose ketamine, both NMDA receptor antagonists known to limit SD propagation. Her facial weakness improved, though speech abnormalities persisted initially. Continuous video EEG showed no epileptiform activity. The patient’s husband noted that these symptoms closely resembled those she had experienced two years prior. By postoperative day (POD) 7, her speech and facial weakness had fully resolved, and the ECoG electrode was removed. A postoperative MRI demonstrated expected postoperative changes at the bypass anastomosis site, with several small punctate deep white matter strokes but no territorial infarct (Figure 1C).

## 3. Discussion

SDs are waves of cortical electrical activity disruption that propagate across brain tissue. Originally described in migraine aura, SDs have been increasingly recognized as key pathophysiological phenomena in various acute and chronic neurological disorders.(4) While typically transient and variable, SDs can induce complex neural network disruptions, leading to regional metabolic stress and reversible neurological dysfunction.(5)

In this case, we observed a real-time temporal correlation between SDs and the patient’s neurological deficits, suggesting that SDs may have contributed to her symptoms. The patient’s initial diagnosis of FND underscores the challenges in differentiating functional disorders from transient neurophysiological disturbances.(6, 7) Notably, her symptoms repeatedly occurred in the absence of new significant structural abnormalities on imaging, underscoring the diagnostic challenge posed by transient yet disabling deficits that leave no radiographic trace. The marked improvement in her symptoms with SD inhibitors such as memantine and ketamine further supports the hypothesis that SDs may be an unrecognized pathophysiological mechanism in a subset of patients diagnosed with FND. Additionally, it is plausible that SDs may underlie FND symptoms by causing transient yet significant network dysfunction.

Current treatment modalities for FND target multiple mechanisms, including cognitive, psychological, and neurophysiological processes. Cognitive behavioral therapy (CBT) remains the primary treatment approach, aimed at addressing maladaptive thought patterns and promoting symptom self-management. Physical therapy, often incorporating techniques such as graded motor imagery and sensory retraining, is beneficial for motor symptoms. Emerging neuromodulatory approaches, including transcranial magnetic stimulation (TMS), have shown promise in modulating dysfunctional brain networks implicated in FND (35074803). Pharmacological interventions, though not first-line, focus on symptom management, particularly in cases with comorbid mood disorders or pain syndromes.

Our findings suggest a novel potential treatment target in SD modulation. If SDs play a role in FND pathophysiology, interventions aimed at preventing or mitigating SD, such as NMDA receptor antagonists (e.g., memantine, ketamine), neuroprotective agents, or targeted neuromodulation, could represent new therapeutic strategies similar to promising results demonstrated in stroke and TBI.(8–10) While there is a paucity of data on the use of memantine in FND treatment, some studies have demonstrated the efficacy of ketamine in treating FND-associated symptoms.(11–13) This case emphasizes the need for further research to explore SDs in functional disorders and assess whether SD-targeting interventions could improve clinical outcomes in select patients.

While causality cannot be established in this report, our findings provide compelling evidence for a potential association between SDs and FND-like symptoms; however, several limitations must be acknowledged. First, this is a single case report, and broader studies are needed to establish a causal relationship. Second, the presence of ICA stenosis introduces a potential confounder, as chronic hypoperfusion could theoretically predispose the cortex to SDs. However, the absence of new infarcts, patency of the bypass, and the reversibility of symptoms argue against an ischemic etiology. Finally, SD monitoring in clinical settings remains limited, and real-time detection outside of intraoperative environments is challenging. Scalp EEG has been investigated as a non-invasive tool for SD detection, but its limited spatial resolution and sensitivity make it difficult to capture key SD features reliably. This limitation is one reason we used intraoperative electrocorticography (ECoG) in this case, which offers direct cortical access and higher fidelity in detecting SDs.(14, 15).

In summary, FND is increasingly understood as a disorder of network dysfunction involving circuits responsible for emotion regulation, attention, interoception, and agency. Disruptions in predictive processing, where expectations dominate over sensory input, may contribute to symptom generation. Within this framework, SDs could represent transient physiological disturbances that trigger or amplify functional symptoms in susceptible individuals due to disruption of complex network activity. Future research utilizing non-invasive neurophysiological tools may help clarify the role of SDs in FND and other functional disorders.

## 4. Conclusion

FND is the second most common reason for neurological consultation in the outpatient setting, with a prevalence of 50–100 cases in 100,000 people.(1) We propose that SDs may contribute to the pathophysiology of symptoms in a subset of patients diagnosed with FND. Recognizing SDs as a potential mechanism may refine diagnostic approaches, encourage consideration of SDs in differential diagnoses, and open new avenues for targeted therapeutic interventions. Further research is warranted to elucidate this potential link and its implications for the broader understanding of FND.

## Figures and Tables

**Figure 1.** Evidence of Spreading Depolarizations (SDs) Following Superficial Temporal Artery to Middle Cerebral Artery (STA-MCA) Bypass. (A) Schematic illustration of the STA (red) to MCA (brown) bypass procedure with electrocorticography (ECoG) electrodes placed over the cortex for postoperative monitoring. Right panel: ECoG recordings filtered at 0.5–50 Hz (black trace) show characteristic spreading depolarizations (SDs) as a slow direct current (DC) shift (red trace) followed by a period of cortical depression (green bars). The onset of SDs coincides with the development of left facial droop and aphasia. (B) Postoperative computed tomography angiography (CTA) demonstrating a patent STA-MCA bypass anastomosis (red circle), ruling out vascular occlusion as a cause of neurological symptoms. (C) Diffusion-weighted magnetic resonance imaging (MRI) obtained postoperatively showing no evidence of large territorial infarction, confirming the absence of ischemic stroke as an alternative explanation for the observed symptoms.

## Learning Points

1. Spreading depolarizations (SDs) can induce transient neurological symptoms and may represent an unrecognized mechanism in patients diagnosed with functional neurological disorder (FND).
2. Intraoperative electrocorticography (ECoG) can identify SDs in real-time, offering insight into unexplained, fluctuating postoperative neurological deficits.
3. Treatment with SD inhibitors such as memantine and ketamine may lead to clinical improvement in patients experiencing SD-associated symptoms.
4. Clinicians should consider SDs in the differential diagnosis of FND, particularly in patients with vascular risk factors or recurrent, unexplained neurological symptoms.
